# No evidence of whole population mental health impact of the Triple P parenting programme: findings from a routine dataset

**DOI:** 10.1186/s12887-017-0800-5

**Published:** 2017-01-31

**Authors:** Louise Marryat, Lucy Thompson, Philip Wilson

**Affiliations:** 1 The Scottish Collaboration for Public Health Research and Policy, University of Edinburgh, 20 West Richmond Street, Edinburgh, EH8 9DX UK; 2 Institute of Health and Wellbeing, University of Glasgow, Caledonia House, Royal Hospital for Sick Children, Yorkhill, Glasgow, G3 8SJ UK; 3 Centre for Rural Health, University of Aberdeen, Centre for Health Sciences, Old Perth Road, Inverness, IV2 3JH UK

**Keywords:** Parenting, Public health, Child psychology, Behavioural family intervention, Observational study

## Abstract

**Background:**

The Triple P parenting programme has been reported to improve child mental health at population level, but it consumes substantial resources. Previous published work has suggested improvements in whole population scores in the Strengths and Difficulties Questionnaire (SDQ) Total Difficulties Scale among samples of children following introduction of the programme. This paper aims to explore whether Triple P had an impact on child mental health problems using routinely collected data over 6 years before and during the implementation of the multilevel Triple P programme in Glasgow City.

**Methods:**

Annual monitoring of teacher-rated SDQ Total Difficulties Scale scores among children in their pre-school year in Glasgow City.

**Results:**

No significant or consistent changes in SDQ Total Difficulties Scale scores were seen during or after the implementation of Triple P programme on a whole population level.

**Conclusion:**

Triple P in Glasgow City appears to have had no impact on early child mental health problems over a 6 year period. The Triple P programme, implemented on a whole population level, is unlikely to produce measurable benefits in terms of child mental health.

**Electronic supplementary material:**

The online version of this article (doi:10.1186/s12887-017-0800-5) contains supplementary material, which is available to authorized users.

## Background

### Rationale

Public policy has increasingly acknowledged the importance of effective parenting as a determinant of population health, and evidence-based parenting programmes have been advocated as a means to reduce societal health inequalities [1–3]. The Positive Parenting Programme (Triple P) [4] is a multi-level behavioural family intervention which has been designed [5, 6] and used [7–12] on a whole-population basis as a public health intervention, in addition to its use with targeted groups. Many administrative entities throughout the world have adopted the programme on a large scale [13].

Triple P has a fairly extensive evidence base, with over 500 publications including a large number of randomised trials. There are seven current meta-analyses of the programme [14–20], demonstrating consistent positive effects on child behaviour. Some doubt has been expressed about the effectiveness of Triple P in deprived communities [16], with lone parents [21] and among younger children. Furthermore, the impact out with these targeted groups has not been explored comprehensively. A substantial amount of the published evidence was conducted by affiliates of the Triple P organization [18].

The Triple P programme intervenes with parents rather than with children or whole families, and the great majority of published outcomes are reported by parents. Child-based outcomes are clearly of primary interest since the parenting programmes aim principally to improve children’s wellbeing, but it can be difficult to differentiate between parents’ mental state and their perceptions of their children’s behaviour or the effectiveness of treatment [22, 23]. Fathers are less likely to attend Triple P parenting programmes than mothers and they are less likely to report improvements in child behaviour [18, 19], while teachers and other independent observers have generally not reported positive effects of Triple P in the relatively small number of published trials for which data are available [18]. The most recent Triple P meta-analysis published by the developers of the programme [19] did not report teacher data.

Two large studies of the whole-population impact of Triple P have examined the mental health of samples of children using the parent-report version of the Strengths and Difficulties Questionnaire (SDQ) [24] before and after implementation [8, 11]. Both these studies used a quasi-experimental design with different samples selected before and after the intervention, and both are limited by substantial baseline differences between the study groups. Nevertheless, significantly better improvements in the SDQ Total Difficulties Scale and the Emotional Symptoms subscale scores were reported in intervention areas compared with control areas in both studies. The current study adds to the evidence base on Triple P through assessing the impact of the whole-population Triple P implementation through an examination of routine data collected through schools on children’s social, emotional and behavioural development at age 4–5, between 2010 and 2015.

#### The Glasgow parenting support strategy

Glasgow City Council and NHS Greater Glasgow and Clyde made a commitment to city-wide implementation of the multilevel Triple P programme in August 2009 [25] and the population roll-out of the programme was officially launched in late May 2010, continuing until 2014. A small number of Triple P groups had been delivered in the city prior to the launch [26] but it is highly unlikely that this activity could have affected baseline data to any significant extent. An evaluation of this complex intervention [27] took place between 2011 and 2014. The process of implementation is reported in detail in the evaluation final report [26], but 730 practitioners were trained in Triple P interventions and over 30,000 Triple P interventions at different levels were delivered between 2009 and December 2013:

- single interventions (one-off interventions such as giving and discussing ‘tip sheets’ on a specific topic, e.g. sleeping) to 12,432 families;
- seminars (generally a one-off seminar primarily delivered during the preschool year) to 13,645 families;
- Primary Care Triple P (a course delivered on a one-to-one basis) to 2527 families;
- Group Triple P (a group-based course) to 2144 families;
- Mass media campaigns (through television, posters and newspapers) aimed at the whole population.

Aside from the media campaigns, this equates to 30,748 families in Glasgow City receiving some form of intervention (though it should be noted that families could receive more than one type of intervention) out of an estimated 57,000 eligible families in Glasgow City [26]. Fewer than half of families that started Primary Care and Group Triple P interventions, which ran over several sessions, completed the programmes. Whilst numbers of families starting interventions fell over the 3 years of implementation being evaluated (Primary Care uptake from 935 to 497 and Group uptake from 995 to 487), rates of completion increased: for Primary Care from 27.2% in year 1, to 51.1% in year 2, to 70.6% in year 3 of implementation; for Group Triple P the equivalent figures were 48.6, 32.6 and 57.7%. Overall, the intervention delivery rates were substantially greater than those reported in the two previous published papers that have reported benefit from whole-population implementation of Triple P [8, 28].

In general, families with children with greater problems (as assessed with the SDQ) compared with families in the general population were more likely to start participating in Group Triple P sessions, but they were also less likely to complete such interventions. Similar patterns could be seen for those in the lowest income households, and those with the lowest levels of education. For more information on implementation, reach and uptake, see the final evaluation report [26].

This paper explores whether any change can be seen in the levels of mental health problems in the population of preschool children (aged 4–5) in Glasgow City during the implementation of Triple P. This is a good age to explore potential impact as it is a time when most children are still spending most of their time with parents, but also when the majority of children attend nursery (kindergarten) for some time each weekday, allowing any mental health problems to be independently assessed by a worker who knows them relative well. Triple P also targeted the preschool age group, and we know from intervention-level data that almost 60% of children for whom the parent stated they were attending a group intervention were aged 0–5 [29].

#### Objectives

We aimed to assess the impact of the whole-population Triple P implementation through an examination of the temporal trends in the mental health of pre-school children between 2010 and 2015.

#### Research questions

1. What is the prevalence of teacher-rated social, emotional and behavioural difficulties in preschool children in Glasgow City and how has this changed following the implementation of Triple P?
2. Do trends differ for children from different socio-economic backgrounds?
3. What factors independently predict higher levels of difficulties?

## Methods

### Protocol and registration

The protocol for this study was published in 2010 [28].

### Study subjects

Pre-school education staff were asked to complete questionnaires on all children progressing to school from a local authority or partnership (private nurseries with places funded by the Local Authority) pre-school establishment in Glasgow City between 2010 and 2015. In the academic year of 2014/15, 90.9% of eligible children (i.e. all children in their preschool year – due to start school in August 2015) in Glasgow City were registered for a preschool place and were thus eligible for inclusion in the study. That year, once the response rate is taken into account, SDQs were available for 81.6% of the population of pre-schoolers in Glasgow City [30] (Table 1).

**Table 1:** Response rates by year of data collection

	2010	2011	2012	2013	2014	2015
Number of complete SDQs	3423	3407	4011	5045	6009	6013
Response rate	67.4	69.3	66.8	85.3	95.7	89.8
Number of nurseries returning data	126	141	101	168	188	189

### Data collection and tools

To assess temporal trends in child mental health, we used scores from the teacher- rated version of the SDQ [24] (Additional file 1) which indicates the likelihood of difficulties in five areas: emotional symptoms, conduct problems, hyperactivity/inattention, peer relationship problems, and prosocial behaviour. As well as five subscale scores, each case has a ‘total difficulties’ score based on the summing of scores on the first four subscales (i.e., all but prosocial behaviour, which is positively scored). Scores can range from 0 to 40, with a threshold of 16 and above on the total difficulties scale suggesting that a child may have a psychiatric diagnosis and/or may require further assessment and support. The SDQ was completed between February and April every year between 2010 and 2015 by preschool education staff for children in Glasgow City during their pre-school year (i.e., between the ages of 4.5 and 5.5 years) as part of routine transition documentation. This meant that the first wave of data collection occurred shortly before the launch of population-wide Triple P in Glasgow City.

Preschool staff were given training in groups by researchers (with top-up training available each year for new staff), as well as written guidance, on completing the SDQ. Whilst in some nurseries the child development officer (CDO, key worker) for the child completed the SDQ alone, in other nurseries the CDO completed the SDQ alongside the nursery head and/or other staff [31]. Inter-rater reliability could not be assessed.

Response rates have grown year on year: from 67.4% of pupils in 2010 to 95.7% of pupils in 2014 and 89.8% in 2015, based on the Scottish Government pupil census. Overall, 224 preschools completed SDQs for at least one time point, while 46 preschool establishments completed data every year. In order to assess whether nurseries that returned data in the early years were systematically different from those who only returned data later, we completed analyses both on all available data and the limited sample of 46 nurseries, and demonstrated minimal differences in trends.

Each child had a SDQ Total Difficulties score and constituent domains recorded along with a code for the pre-school establishment that they attended. In 2010, data were completed using paper questionnaires, whilst in 2011 a mixture of paper and electronic forms were completed, moving to solely electronic completion by 2012. These data were linked to data held in the education services database comprising age, gender and deprivation status of the locality in which the child lived.

Two versions of the SDQ are available for this age group: a version for 3–4 year olds and one for 4–16 year olds. The 4–16 version was used in 2010 and 2011, but changed to the 3–4 version in 2012, following interviews with pre-school staff [29]. The difference between the two versions is relatively small and involves the wording of two questions in the 4–16 conduct scale, about lying, cheating and stealing, which were changed to slightly ‘softer’ and more age appropriate questions about being argumentative with adults and being spiteful. Staff may have more readily answered positively to these softer questions in 2012 and thereafter [29]. For this reason analyses conducted in this report use the shorter version of the conduct problems scale using only the same three questions which were asked every year. A score was then calculated in the usual way for SDQ, though using three answers rather than four or five: the three questions were used to give an average score and then multiplied by five in the usual way. Thus the same cut-offs for Conduct Problems were used. Cronbach’s alpha for the 5-item Conduct Problems scale in the present sample was 0.72. This fell slightly to 0.66 when it was reduced to three items. A score of 0.70 or above is usually deemed acceptable, although scores are partly related to the number of items in the scale as well as their internal consistency [32].

Since information on the socioeconomic status of individual children (e.g. household income or occupational social class of head of the household) was not available, deprivation status was based on the postcode of the children’s home address and measured using the Scottish Index of Multiple Deprivation (SIMD). For each postcode, the publicly available SIMD was obtained. The SIMD is a continuous measure of compound social and material deprivation, calculated using income, employment, health, education, housing, geographic access to services and crime data [33]. The measure is updated approximately every 4 years. The current analysis used the 2009 SIMD measure. SIMD is normally banded into quintiles. Due to the skew of the data in Glasgow City, whereby around 50% of people fall into the ‘most deprived’ SIMD quintile, Glasgow City have developed their own quintiles (GIMD quintiles), which allow for more in-depth analysis of deprivation within the city, and it is these which are used in these analyses [33].

### Analysis

Annual trends for SDQ total difficulties scale scores are presented, together with data trends by levels of deprivation of the children.

In order to reduce the risk of bias associated with different pre-school establishments (and thus different groups of staff) completing SDQ data in successive years, trends were plotted for the subgroup of these establishments which returned data in each year 2010–2015. Both continuous scores and banded scores were included in the analyses in recognition of the hypothesis that, although we are primarily interested in children moving out of the abnormal range, there may have been improvements made within the normal range in this whole population sample. Results for the conduct problems subscale are presented using only the 3-question version of the scale which was the same every year.

Tests for change over time were ANOVA tests and Post Hoc multiple comparisons in the form of Tukey tests. A multilevel approach (using child and nursery as levels) was explored but rejected due to the lack of difference between levels.

Linear regression models were fitted in order to investigate predictors of total difficulties scores. Variables entered into the models were cohort, sex, ethnic status, Looked After status, and GIMD.

### Ethics

Formal ethical review board approval was not required for the present analysis of the anonymised dataset, but the ethical issues possibly raised by this study were considered by the research team. It was concluded that the project posed no harm to the participants, the schools or the different regions, as the anonymized data were collected by educational establishments as part of the routine documentation passed to primary schools for the benefit of teachers and pupils. A data sharing agreement was in place between Glasgow City Council and the University of Glasgow to facilitate the safe sharing of data.

### Consent

Parents were sent information about the study through their child’s nursery and were able to opt out of the data collection on behalf of their child. Education services handled the opt-outs and have reported that very few (i.e., less than 5 per year) were received.

## Results

The sample comprised 51.3% boys and 48.7% girls: this did not differ by year. Ethnic minority groups ranged from 26 to 31.9%, though there was no discernible pattern in these differences by year. The number of children recorded as having ever been Looked After (i.e. in the care of the local authority) has fallen over the years from 2.1% in 2010 and 3.1% in 2011, to 0.4% in 2015. It is clear, following consultation with Glasgow City Education Services, that this is likely due to changes to reporting and/or recording of Looked After Status in the latter years rather than a true change in prevalence. This view is supported by recently published Scottish Government statistics showing that 3.02% of children in Glasgow City were officially recorded as being ‘Looked After’ in 2014 [34]. Levels of children living in neighbourhoods with the highest levels of deprivation varied slightly from year to year (26.1 to 32.8%), with higher levels of children living in the most deprived areas in 2011 and 2012. When restricted to the same preschool establishments each year, the proportion of children in the most deprived areas is slightly higher (from 31.6 to 34.8%), probably due to private preschools (more often used by wealthier families) being under-represented in the early years of data collection, and increased funding eligibility (and hence social inclusiveness) of private preschool establishments when contracts were renewed by the city council in 2014.

**Fig. 1:**
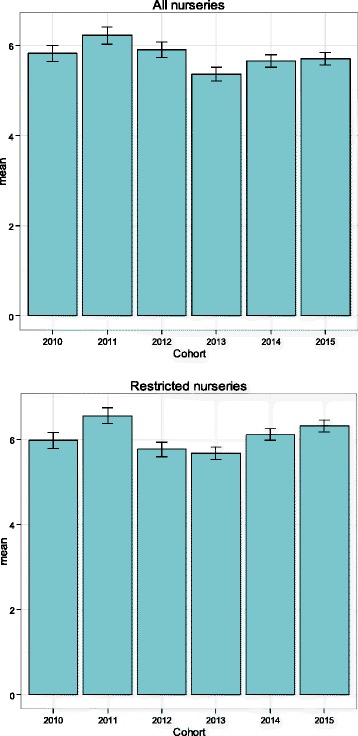
Mean SDQ scores (with 95% confidence intervals) by year of cohort (*top*: all preschools; *bottom*: restricted to preschools which returned data every year)

Mean scores were explored first. Mean total difficulties scores showed little variation across years whether examining the total cohort or restricting analyses to those nurseries which had returned data every year. A corresponding lack of any discernible trend was found across all subscales, with minor fluctuations in mean scores being evident. These data are illustrated in Fig. 1.

Mean scores across the years were also explored by deprivation quintile. Children in the least deprived areas had lower mean scores than children in all other areas at every time point, with no discernible trends in terms of total difficulties scores across the years found in any deprivation group (Additional file 2: Figure S2).

In order to check whether significant differences existed between years, ANOVA tests were completed and Post Hoc multiple comparisons in the form of Tukey tests were explored. Initial ANOVA tests on the full dataset (all nurseries) demonstrated a significant level of variance between years for the Total Difficulties score and all SDQ subscale scores, however no linear patterns were found. Post hoc tests for the Total Difficulties scores suggested that 2011 scores (the second year of data collection) were significantly different to all other years, but no other patterns were observed. On the three item Conduct Problems scale, 2010 was significantly different to all later years, except for 2011, though no other significant differences were observed and no linear patterns could be seen (Additional file 3).

When the tests were repeated for the nurseries which returned data each year, ANOVA once again suggested a significant difference between all years, though again, no linear patterns could be seen. Again on the post-hoc tests, 2011 appeared to be different from almost all other years on the total difficulties scale, whilst 2015 was additionally significantly different from 2012 to 2013. In relation to conduct problems, using the same three-item scale each year, the only significant difference was between 2010 and 2012/2013. Some significant differences could also be found on other subscales, however, there was no pattern to the differences and early scores were generally not found to be significantly different to the later scores (Additional file 4: Table S1).

**Fig. 2:**
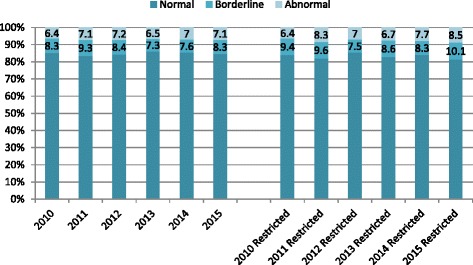
Total difficulties banded scores by year (all nurseries and restricted to preschools which returned data every year)

There was no evidence that children with more severe difficulties were selectively affected. No significant difference could be seen across the years in the proportion of children with abnormal scores, which ranged from 6.4 to 7.2% for the Total Difficulties Scale (Fig. 2). When analysis was restricted to the nurseries that returned data each year, a similar pattern was seen. Proportions of scores in the abnormal or borderline range for each of the SDQ subscales are illustrated in the online supporting document. No significant changes were seen over time in any subscale.

Linear regression models were fitted to explore associations with Total Difficulties scores. The first model, using all available data, showed that being male, having ever been ‘Looked After’ by the local authority, and being of a white UK ethnicity was associated with a higher total difficulties score in preschool, whilst living in a less deprived area was associated with a lower score. When the models were restricted to data from nurseries which had returned data every year, results were very similar, though ethnicity was no longer significantly associated. No cohort year was significantly associated with the Total Difficulties score (Table 2).

**Table 2:** Linear regression models predicting total difficulties scores in preschool

	Model 1 – all nurseries	Model 2 – restricted to same nurseries
	β standardized	β standardized
*Sex*		
Female	0	0
Male	0.199**	0.202**
*Looked After Status*		
Never Looked After	0	0
Ever Looked After	0.084**	0.073**
*Ethnic status*		
Non-UK White	0	NS
UK White	0.018*	
*GIMD*		
Less Deprived	-0.091**	-0.108**
*Rsq*	*0.056*	*0.059*

Entered into model: cohort year, sex, Looked after status, Ethnic status, multiple deprivation quintile (GIMD)

*P* value: <0.01**; <0.05*

## Discussion

### Main findings

No significant improvement was found in the social, emotional and behavioural difficulties of preschool aged children between 2010 and 2015, suggesting that Triple P had no impact on population-level mental health problems in children in Glasgow City during this period. This conclusion was the same whether examining mean scores or banded abnormal scores, and whether analysing all data collected or restricting analysis to data from the nurseries which returned data each year, and dependent on different levels of deprivation experienced by the children. Across all areas of difficulties, scores fluctuated slightly from year to year but did not indicate any significant improvement.

The strongest predictor of poorer mental health at preschool was being male, in line with previous evidence around child mental health. [35, 36] There is evidence to suggest that a reporting bias in both teachers and parents whereby problems in boys are over-reported due to an overly negative view of boys’ behaviour [37]. In addition, being in a more affluent area was associated with having lower levels of mental health difficulties, whilst having been under the supervision of the state (‘Looked After’) was associated with having higher levels of difficulties, both findings in line with previous reports [38]. Being of a white UK origin was associated with poorer mental health at preschool, though the significance of this disappeared once analysis was restricted to the same nurseries each year, suggesting that this finding may be an artefact of the sampling. Previous findings in relation to ethnicity in the UK and childhood mental illness have been mixed [38–40].

Implementation is key to the success of any intervention [41]. Although reach was good, in terms of parents with the greatest need starting interventions, fewer than half of families completed Group and Primary Care interventions, with those from more deprived areas and those with more difficulties being less likely to complete. This was in spite of a substantial investment by staff to increase engagement as much as possible, including the provision of childcare, a range of times/locations of interventions, and using the flexibility allowed within the Triple P system (e.g., adapting language to more colloquial terms). Intervention completion rates have not been reported in previous whole population studies of Triple P. Whilst low completion rates may account in part for the lack of impact of Triple P in Glasgow, this may also indicate that it may not have been an appropriate choice of intervention for this population. It is equally possible that Triple P interventions, even when they were completed, did not affect the mental health problems observed by nursery staff.

### Strengths

This unique large population-level dataset has many strengths for an assessment of the impact of a public health approach to improving childhood social and behavioural outcomes. The dataset covers a very large proportion of the population: around 91% of children were eligible and response rates were up to 96%. Furthermore, the sample has excellent representation of children from the most deprived areas, which is often lacking in other sample-based studies.

The teacher version of the SDQ was filled out by preschool staff, which offers a level of objectivity to the data. Many evaluations and cohort studies in this field (including the vast majority of Triple P evaluations) rely on parent-reported measures, which may be affected by bias related to potentially transient improvements in parental mental state following intervention [18]: previous research indicates that mothers experiencing depression are more likely to view their children’s behaviour as problematic [42]. Preschool staff also have a view of what is ‘normal’ behaviour at each age.

Six years of data were available, spanning the period of implementation of Triple P and beyond. In the context of prior claims about the efficacy of the population impact of Triple P, [7–9, 11, 12] this should give sufficient time, both to identify trends and for Triple P to be embedded into the public health landscape.

The two population studies reporting improvement in SDQ total difficulties and emotional symptoms scales used a design involving interviews with large but different samples pre- and post-intervention. [8, 11] Our analysis restricted to those nurseries which returned data in each year of the evaluation largely addresses this issue of inconsistency of informants.

### Weaknesses

There are also some weaknesses to the study. Perhaps most importantly, there is no comparison group; it is possible that scores might have got worse without Triple P, though this is thought unlikely given general lack of these trends being reported over time in, for example, previous UK national surveys [35, 39]. Existing quasi experimental studies and population trials have claimed differences over time but interpretation is rendered difficult by substantial problems with sampling strategy and other design features [18].

Only data for preschool aged children (aged 4–5 years) were explored. Although many families with children this age will have been exposed to Triple P, either through the cohort child or their siblings, it is impossible to specify how many, and which, of the families of these cohort children were directly exposed to the intervention or to population level media campaigns. The intensity of intervention in relation to the population size was at least as high with that reported in previous studies reporting positive results [7, 8].

Although teacher-rated SDQs provide a more objective rating of the child’s difficulties, the addition of parent-rated SDQs would have enhanced the study and possibly contributed to an understanding of the discrepancy between our negative findings and those reported by the developers of Triple P. Parent scores were not routinely collected in our study because of a lack of resources. The use of multiple informants has been shown to give better predictive values for diagnosis [43, 44] but response rates from parents in population studies of this type would inevitably be lower and biased towards more affluent and educated parents, as well as towards children with fewer difficulties [44–46]. Furthermore, only child outcomes were explored. It may be that Triple P is successful in building parents’ confidence or achieving other parental outcomes, but we have reported on the objective impact on the population of children, which we consider more important as a public health outcome.

### Comparison with existing literature

The results from this study suggest that Triple P did not make a difference to population levels of mental health problems in Glasgow City preschool children over a 6 year period. This contrasts with the published whole-population studies. The South Carolina study [45] was a cluster randomized trial, but although data were collected on child mental health, [9] the published data only relate to maltreatment rates. Three other population-level Triple P evaluations which include a control group exist, all reporting positive results of the intervention. Sanders et al [8] conducted a quasi-experimental evaluation in three Australian cities. Substantial differences could be seen at baseline between the intervention and control groups. Around 3000 parents were assessed before and after the intervention, but different samples were used at each time point, making it impossible to examine changes in individuals. Only the proportion of children with “clinically elevated” scores was reported, as opposed to mean or median scores. A more recent study carried out by Fives and colleagues in Ireland [11] followed this study design closely and obtained very similar results: reductions in SDQ total difficulties and emotional symptoms subscale scores.

Zubrick et al [12] conducted another quasi-experimental evaluation, again in Western Australia. Once more, substantial differences could be found between the intervention and control groups at baseline. Importantly, families were recruited differently for each group: whereas intervention group parents volunteered to participate in a group, control group parents volunteered only to participate in a survey of child behaviour. Analysis using hierarchical linear modelling suggested an improvement in ECBI externalizing behaviour scores in the short-term, however, given the potential for confounding due to the differential recruitment, it is difficult attributable this causation solely to the intervention.

Furthermore, a recent cluster randomised control trial exploring the impact of Triple P levels 2 and 3 on pre-schoolers’ externalising behaviours and parental mental health concluded that there was no evidence that either externalising behaviours in the children or mental health in parents was improved by attending the intervention [47, 48].

It could be that the difference between parental and teacher reports of child behaviour reported in the literature [18] may be related to the potential for the maternal mental state to improve, at least transiently, with the majority of Triple-P interventions [15, 19] which may in turn lead to a more positive assessment of the child’s behaviour, reflecting a greater degree of optimism. It nevertheless remains conceivable that mothers may more accurately report their children’s than others. Notably however, one study [21] reported an intended subgroup analysis focusing on lone parent families – and findings showed no benefit from the Triple P intervention. Most participants in published Triple P trials have been well-educated and married or in stable relationships [18] in contrast to the demographic composition of the participants in interventions in the Glasgow Parenting Support Framework, [26] and it is possible that this difference underlies some of the difference between our results and previous reports.

The lack of convincing evidence of benefit from the Glasgow City whole-population intervention is, however, in line with other previous work in which no significant improvement in child-based outcomes resulted from a public health parenting programme [49] and with the latest independent randomised trials of Triple P, which indicated that there was no impact on child behaviour [50–53].

## Conclusions

Claims that parenting programmes which focus on the whole-population demonstrate significant impact on the health of the population are especially important, because these might have resulted in a substantial commitment of public funds. No convincing evidence of benefit for preschool aged children’s mental health problems from the Triple P programme in the whole-population implementation in Glasgow was found in the current study.

## Additional files

Additional file 1: SDQ Questionnaire. PDF (PDF 108 kb)
Additional file 2: Figure S2. Mean Total Difficulties score by Glasgow Deprivation Quintile and Year. PDF (PDF 238 kb)
Additional file 3: Figure S1. Proportion of children with abnormal scores on the SDQ subscales by year. PDF (PDF 180 kb)
Additional file 4: Table S1. Post hoc Tukey test results for Total Difficulties scores by cohort year. PDF (PDF 56 kb)

## Abbreviations

GCES: Glasgow council education services
GIMD: Glasgow index of multiple deprivation
NHS: National health service
SDQ: Strengths and difficulties questionnaire
SIMD: Scottish index of multiple deprivation
Triple P: The positive parenting program
