# Supplementary material for: No evidence of whole population mental health impact of the Triple P parenting programme: findings from a routine dataset
Source: BMC Pediatr. 2017 Jan 31;17:40. doi: 10.1186/s12887-017-0800-5 (PMC5282654; doi:10.1186/s12887-017-0800-5)
Supplement: Additional file 1: — SDQ Questionnaire. PDF (PDF 108 kb) [file 12887_2017_800_MOESM1_ESM.pdf]

Strengths and Difficulties Questionnaire

For each item, please mark the box for Not True, Somewhat True or Certainly True. It would help us if you answered all items as best you can even if you are not absolutely certain or the item seems daft! Please give your answers on the basis of the child's behaviour over the last six months or this school year.

Child's Name .... Male/Female

Date of Birth...

	Not True	Somewhat True	Certainly True
Considerate of other people's feelings	☐	☐	☐
Restless, overactive, cannot stay still for long	☐	☐	☐
Often complains of headaches, stomach-aches or sickness	☐	☐	☐
Shares readily with other children (treats, toys, pencils etc.)	☐	☐	☐
Often has temper tantrums or hot tempers	☐	☐	☐
Rather solitary, tends to play alone	☐	☐	☐
Generally obedient, usually does what adults request	☐	☐	☐
Many worries, often seems worried	☐	☐	☐
Helpful if someone is hurt, upset or feeling ill	☐	☐	☐
Constantly fidgeting or squirming	☐	☐	☐
Has at least one good friend	☐	☐	☐
Often fights with other children or bullies them	☐	☐	☐
Often unhappy, down-hearted or tearful	☐	☐	☐
Generally liked by other children	☐	☐	☐
Easily distracted, concentration wanders	☐	☐	☐
Nervous or clingy in new situations, easily loses confidence	☐	☐	☐
Kind to younger children	☐	☐	☐
Often argumentative with adults	☐	☐	☐
Picked on or bullied by other children	☐	☐	☐
Often volunteers to help others (parents, teachers, other children)	☐	☐	☐
Can stop and think things out before acting	☐	☐	☐
Can be spiteful to others	☐	☐	☐
Gets on better with adults than with other children	☐	☐	☐
Many fears, easily scared	☐	☐	☐
Sees tasks through to the end, good attention span	☐	☐	☐

Signature .... Date ....

Parent/Playgroup leader/Nursery teacher/Other (please specify:)

Thank you very much for your help

© Robert Goodman, 2005
