# Supplementary material for: No evidence of whole population mental health impact of the Triple P parenting programme: findings from a routine dataset
Source: BMC Pediatr. 2017 Jan 31;17:40. doi: 10.1186/s12887-017-0800-5 (PMC5282654; doi:10.1186/s12887-017-0800-5)
Supplement: Additional file 2: Figure S2. — Mean Total Difficulties score by Glasgow Deprivation Quintile and Year. PDF (PDF 238 kb) [file 12887_2017_800_MOESM2_ESM.pdf]

Additional file 2: Figure S2 Mean Total Difficulties score by Glasgow Deprivation Quintile and Year

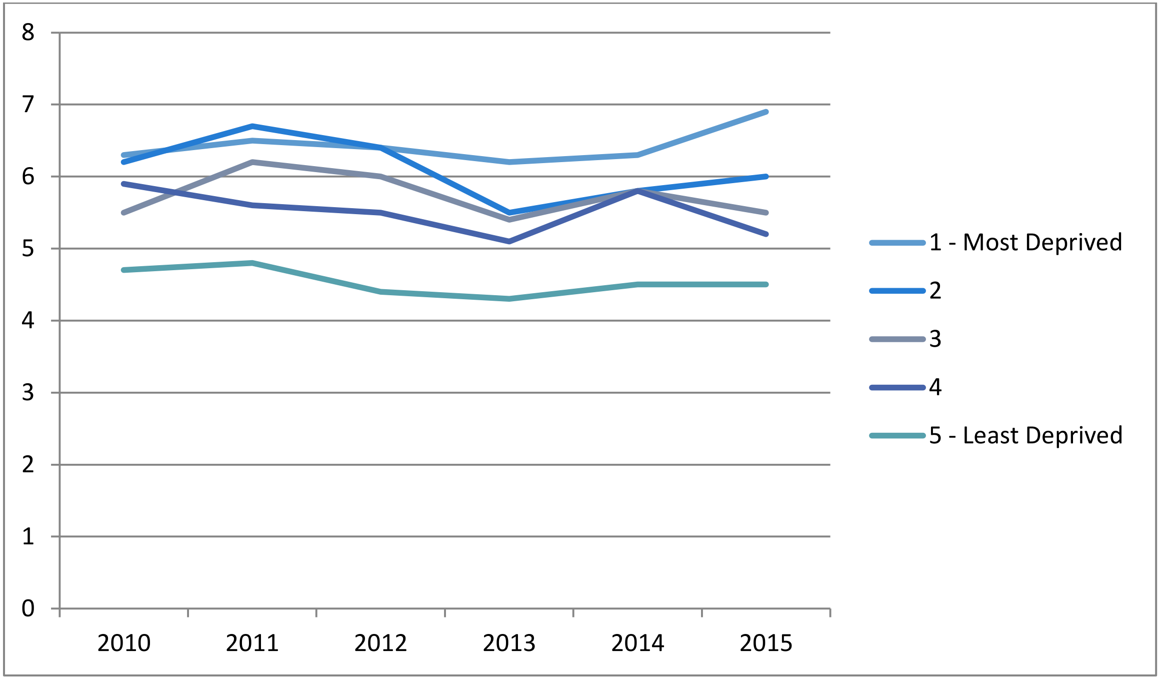

Base: 26,156
