# Supplementary material for: No evidence of whole population mental health impact of the Triple P parenting programme: findings from a routine dataset
Source: BMC Pediatr. 2017 Jan 31;17:40. doi: 10.1186/s12887-017-0800-5 (PMC5282654; doi:10.1186/s12887-017-0800-5)
Supplement: Additional file 3: Figure S1. — Proportion of children with abnormal scores on the SDQ subscales by year. PDF (PDF 180 kb) [file 12887_2017_800_MOESM3_ESM.pdf]

Additional file 3: Figure S1 Proportion of children with abnormal scores on the SDQ subscales by year: top = data from all nurseries; bottom = data from nurseries which returned SDQs every year

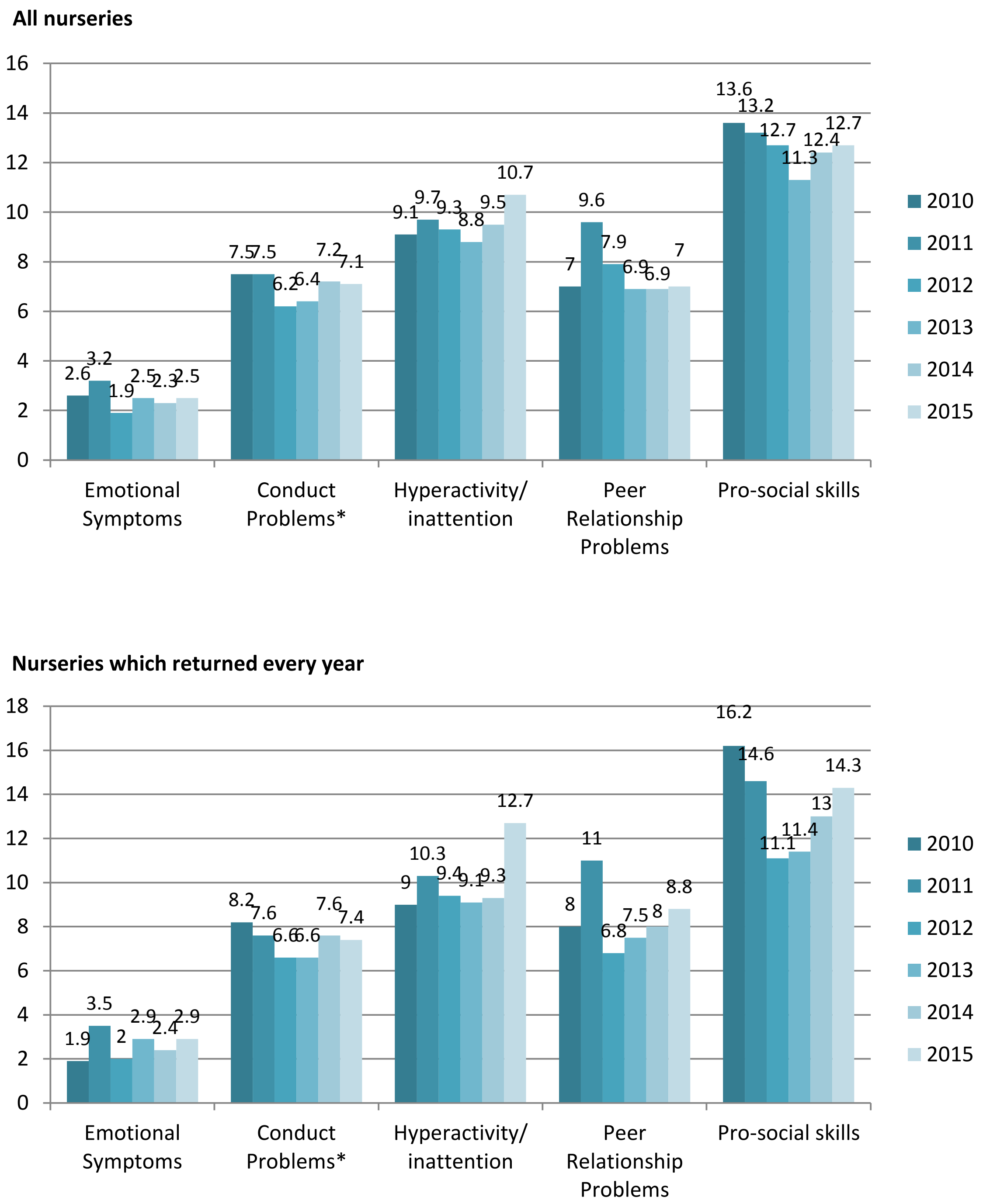

*Bases: 27,908 *Using the 3-question conduct problems version completed every year*
