# Supplementary material for: No evidence of whole population mental health impact of the Triple P parenting programme: findings from a routine dataset
Source: BMC Pediatr. 2017 Jan 31;17:40. doi: 10.1186/s12887-017-0800-5 (PMC5282654; doi:10.1186/s12887-017-0800-5)
Supplement: Additional file 4: Table S1. — Post hoc Tukey test results for Total Difficulties scores by cohort year. PDF (PDF 56 kb) [file 12887_2017_800_MOESM4_ESM.pdf]

Additional file 4: Table S1 – Post hoc Tukey test results for Total Difficulties scores by cohort year

		Mean difference					
		**2010**	**2011**	**2012**	**2013**	**2014**	**2015**
All available data	**2010**		0.404*	0.090	-0.457**	-0.158	-0.114
	**2011**	-0.404*		-0.313	-0.861**	-0.562**	-0.517**
	**2012**	-0.090	0.313		-0.547**	-0.249	-0.204
	**2013**	0.457**	0.861**	0.547**		0.298	0.343*
	**2014**	0.158	0.562**	0.249	-0.298		0.045
	**2015**	0.114	0.517**	0.204	-0.343*	-0.045	
Restricted to nurseries who returned every year	**2010**		0.580*	-0.206	-0.297	0.145	0.346
	**2011**	-0.580*		-0.786**	-0.877**	-0.435	-0.234
	**2012**	0.206	0.786**		-0.091	0.351	0.552*
	**2013**	0.297	0.877**	0.091		0.442	0.643**
	**2014**	-0.145	0.435	-0.351	-0.442		0.201
	**2015**	-0.346	0.234	-0.552*	-0.643**	-0.201	

***indicates significance at p<0.05, ** indicates significance at p<0.01**
